# The extradomain a of fibronectin enhances the efficacy of lipopolysaccharide defective *Salmonella* bacterins as vaccines in mice

**DOI:** 10.1186/1297-9716-43-31

**Published:** 2012-04-19

**Authors:** Beatriz San Román, Victoria Garrido, Pilar-María Muñoz, Laura Arribillaga, Begoña García, Ximena De Andrés, Virginia Zabaleta, Cristina Mansilla, Inmaculada Farrán, Iñigo Lasa, Damián De Andrés, Beatriz Amorena, Juan-José Lasarte, María-Jesús Grilló

**Affiliations:** 1 Instituto de Agrobiotecnología (CSIC-UPNA-Gobierno de Navarra), Carretera de Mutilva, s/n, 31192 Pamplona, Spain; 2 Centro de Investigación y Tecnología Agroalimentaria (CITA) de Aragón (Gobierno de Aragón), Carretera de Montañana, 930, 50059 Zaragoza, Spain; 3 Área de Terapia Génica y Hepatología, Centro de Investigación Médica Aplicada (CIMA), Pío XII, 55, 31008 Pamplona, Spain

## Abstract

The Extradomain A from fibronectin (EDA) has an immunomodulatory role as fusion protein with viral and tumor antigens, but its effect when administered with bacteria has not been assessed. Here, we investigated the adjuvant effect of EDA in mice immunizations against *Salmonella enterica* subspecies *enterica* serovar Enteritidis (*Salmonella* Enteritidis). Since lipopolysaccharide (LPS) is a major virulence factor and the LPS O-polysaccharide (O-PS) is the immunodominant antigen in serological diagnostic tests, *Salmonella* mutants lacking O-PS (rough mutants) represent an interesting approach for developing new vaccines and diagnostic tests to differentiate infected and vaccinated animals (DIVA tests). Here, antigenic preparations (hot-saline extracts and formalin-inactivated bacterins) from two *Salmonella* Enteritidis rough mutants, carrying either intact (SEΔ*waaL*) or deep-defective (SEΔ*gal*) LPS-Core, were used in combination with EDA. Biotinylated bacterins, in particular SEΔ*waaL* bacterin, decorated with EDAvidin (EDA and streptavidin fusion protein) improved the protection conferred by hot-saline or bacterins alone and prevented significantly the virulent infection at least to the levels of live attenuated rough mutants. These findings demonstrate the adjuvant effect of EDAvidin when administered with biotinylated bacterins from *Salmonella* Enteritidis lacking O-PS and the usefulness of BEDA-SEΔ*waaL* as non-live vaccine in the mouse model.

## Introduction

Fibronectin is a dimer, with monomers comprising three types (I, II and III) of homologous repeat units [1]. Three of its alternatively spliced exons (IIIA, IIIB and IIIC, also referred to as EDA -Extra domain A-, EDB -Extra domain B- and III-CS) correspond to the type III repeat unit [2]. EDA and EDB are produced in vivo in response to tissue injury or other warning signals. Particularly, EDA is produced during embryonic development and in rheumatoid arthritis, wound healing, epithelial fibrosis, vascular intimae proliferation or inflammation in adults [3,4]. EDA induces NF-κB factor activation, proteoglycan release, and 1, 2 and 9 metalloproteinase production, involved in connective tissue destruction following lesion development, as well as in monocyte and dendritic cell migration through the basal membrane. Such events are triggered in response to the interaction between EDA and its specific Toll Like Receptor 4 (TLR-4), which also binds to lipopolysaccharide (LPS) of Gram negative bacteria [5,6]. Hence, EDA has been proposed to induce maturation of dendritic cells through TLR-4 activation, favouring antigen uptake, expression of co-stimulatory signals, antigen presentation and induction of anti-viral or anti-tumoral T cell responses [6-8]. Recombinant EDA has been expressed in *E. coli* and tobacco chloroplasts, maintaining its proinflammatory properties [8]. Easy scale-up, high safety standards and an enormous capacity to synthesize and accumulate foreign proteins in plant chloroplasts [9] may be advantages of using plants as production platforms for biopharmaceuticals [10].

Several *Salmonella* live attenuated strains, including auxotrophic, metabolic and structural (semi-rough) attenuated mutants [11-15] have been proposed as effective vaccines for animals. However, these vaccines are often not safe enough in animals, may release genetically modified microorganisms to the environment and human food-chain, and may interfere in serological diagnostic tests based on the detection of antibodies against the LPS O-Polysaccharide (O-PS) [16] and/or flagellin [17], limiting their practical use in sanitary control campaigns. Therefore, most of the currently recommended vaccines against animal salmonellosis include bacterins from smooth attenuated *Salmonella* spp. [18]. Also, bacterial hot-saline (HS) extracts have been proposed as safe vaccines [19,20]. Since LPS is a major virulence factor and the LPS O-PS is the immunodominant antigen in serological diagnostic tests, *Salmonella* mutants lacking LPS O-PS (rough mutants) represent an interesting approach for developing new live attenuated vaccines [13]. In line with this, some rough mutants such as *Salmonella* Typhimurium Δ*rfaH* have been proposed as live vaccine candidates [21,22] but others have been considered either too virulent (such as *Salmonella* Typhimurium Δ*waaL*) to be safe or too attenuated (*Salmonella* Typhimurium Δ*galE* or Δ*waaG* mutants) to confer protection against virulent infections, being discarded as vaccine candidates [20].

This work aims to investigate the adjuvant value of EDA, using HS or bacterins obtained from *Salmonella* Enteritidis rough mutants differing in LPS-Core composition (SEΔ*waaL* and SEΔ*gal*) as antigenic preparations, in a sublethal challenge mouse model.

## Materials and methods

### Bacterial wild-type and mutant strains

Parental wild-type (SE-wt) strain *Salmonella* Enteritidis 3934 [23] was used as parental strain to produce rough mutants, as smooth control strain and as virulent strain for challenge. Mutant strain SEΔ*waaL* was obtained by replacing the *waaL* gene with a chloramphenicol resistance cassette using a one-step inactivation technique [24] with some modifications [25]. The chloramphenicol resistance cassette was amplified by PCR from the *MudQ* transposon, using the *waal-Clo* Fw and *waal-Clo* Rv primers described in Table 1. Mutant strain SEΔ*gal* was constructed by 4378 bp *gal*ETKM operon deletion using the plasmid pKO3blue [26] and the *gal*-A/*gal*-B and *gal*-C/*gal*-D pairs of primers (Table 1).

**Table 1:** Oligonucleotides designed and used in this study

**Oligonucleotide**	**Sequence (5′to 3′)**
*waal-Clo* Fw	TCACCAGAACAGAACCTGGCGAAT TTAGATGCCACAAGC GTATTTGGAAAGATTCATTA AGTGTAGGCTGGAGCTGCTTC^a^
*waal-Clo* Rv	AGTTGGGAAAATGTAGCGCAGCG TTTCGAGGAACAAAT GAAAAACTGGTTTGATAAGTG ACATATGAATATCCTCCTTAG^A^
*gal*A	GCGGCCGCATTCAGCCCCTGCAACG
*gal*B	CTCGAGGCCGCTACATGCCCGA
*gal*C	CTCGAGCTCCGTTAAGCCTATGGT
*gal*D	AGATCTAATCTGGTGACCGACAGA

^a^The primer sequence for the chloramphenicol resistance cassette is underlined.

### Characterization of rough mutants

The absence of LPS O-PS and the differences in size (i.e. in core structure) of the LPS-Core in SEΔ*waaL* and SEΔ*gal* rough mutants were verified by SDS-PAGE silver staining, modified for LPS identification as described previously [27]. Surface topology was analyzed by susceptibility to 17 *Salmonella* Enteritidis Typing Phages (SETPs; named from SETP 1 to 17) and Felix O1 (FO; which specifically recognizes the outer LPS-Core of most *Salmonella* spp.), according to standard protocols [28,29] of the Salmonella National Reference Laboratory (Instituto de Salud Carlos III, Madrid, Spain). The presence of flagellum antigens was determined by direct anti-H slide agglutination tests [18] and motility was analyzed by the halo generated in swimming and swarming assays [30]. The outer membrane permeability of the mutants was assessed by both the Minimal Bactericidal Concentration to Polymyxin B using a standard microdilution test [31,32] and the bactericidal effect of non-immune human serum [33]. Other phenotypic characteristics were assessed as described elsewhere [23]. Finally, virulence studies were performed in BALB/c mice, by determining lethality and splenic infections in surviving mice. For this, mice (*n* = 5) were inoculated intraperitoneally with 2 × 10^3^, 2 × 10^5^ or 2 × 10^7^ CFU/animal of SEΔ*waa*L or SEΔ*gal* mutants. A group of mice (*n* = 5) inoculated with 2 × 10^3^ CFU/mouse of smooth parental SE-wt strain was used as reference. Deaths were recorded for 2 weeks after infection. Then, the percentage of cumulative survival in mice was calculated by the Kaplan-Meier analysis, and statistically compared by the LogRank test (see below). Moreover, the number of viable bacteria was determined in spleens of surviving mice and expressed as the mean ± SD of log_10_ CFU/spleen, as described previously [19].

### Production and characterization of antigenic preparations

Recombinant protein EDA was obtained either in *E. coli* BL21(DE3) cells (Amersham Pharmacia Biotech) or tobacco plant chloroplasts (named MEDA) as described previously [6,8]. After filter-sterilization in a 0.2-μm membrane (Millipore), the absence of bacterial contaminants in both EDA preparations was assessed by plating onto agar.

Bacterial antigens were obtained by HS extraction or formalin-inactivation (bacterins) from SEΔ*gal* (HS-SEΔ*gal* and B-SEΔ*gal*), SEΔ*waaL* (HS-SEΔ*waaL* and B-SEΔ*waaL*) and SE-wt (HS-SEwt and B-SEwt) strains, as described previously [19,34].

To improve binding of EDA to the bacterial cell surface, a recombinant fusion protein of EDA and streptavidin (named EDAvidin) was obtained and mixed with bacterins previously biotinylated to obtain the antigenic preparations named BEDA. EDAvidin was produced from the expression plasmid pET21a-EDA-Streptavidin constructed with the pET21a-Streptavidin-Alive [35] expressing wild-type subunit of streptavidin with a 6His-tag. The DNA sequence encoding EDA was amplified by PCR, using primers CATATGAACATTGATCGCCCTAAAGGACT (Upper EDA-NdeI) and CATATGTGTGGACTGGATTCCAATCAGGGG (Lower EDA-NdeI) and the plasmid pET20b1-2 as a probe. The resulting PCR product was cloned in pCR2.1-TOPO using the TOPO TA Cloning® kit (Invitrogen LifeTechnologies). All constructs were verified by DNA sequencing. The resulting plasmid expressing EDA in the C-terminus of streptavidin was employed for transformation and expression of EDAvidin in *E. coli* BL21(DE3) cells, and the fusion protein was purified by affinity chromatography (HisTrap^TM^ HP columns, GE Healthcare Life Sciences). The Sulfo-NHS-SS-Biotin system (ThermoScientific) was used for bacterin biotinylation, and the non-reacting Sulfo-NHS-SS-Biotin molecules were removed by dialysis using a Slide-a-Lyzer Dialysis cassette (3,500-MWCO, ThermoScientific). To determine the level of free amines, bacterins B-SEΔ*gal*, B-SEΔ*waaL* and B-SEwt were labeled with carboxyfluorescein succinimidyl ester (CFSE) 0.125 μM (Invitrogen), washed twice and analysed by flow cytometry. Unlabeled SE-wt bacterins were used as negative control.

LPS was quantified by detecting 2-keto-3-deoxyoctonate (Kdo) corrected for 2-deoxyaldoses, as described previously [36]. Protein and antigenic profiles of bacterial and recombinant protein preparations were analysed by Coomassie (Bio-Rad) and immunoblotting methods, respectively. Where indicated, samples were also loaded without boiling onto the SDS-polyacrylamide gels in order to visualize the presence of tetramers of the EDAvidin fusion protein. Immunoblotting was performed using sera from mice experimentally infected with smooth SE-wt or from EDA hyperimmunized rabbits as primary antibodies and horseradish anti-mouse IgG or goat anti-rabbit IgG (ThermoFisher) as secondary antibodies, and the reaction was developed with diaminobenzidine. Proteins were quantified by the Bradford method (Bio-Rad).

Finally, the ability of EDAvidin to bind biotinylated and non-biotinylated bacterins was assessed by ELISA in plates coated with 0.1 μg/well of biotinylated bacterins or conventional bacterins as control. After incubation with 10% foetal calf serum (Invitrogen LifeTechnologies), 3 μg/mL of EDA or EDAvidin proteins were added, incubated with anti-EDA rabbit polyclonal antibody and anti-rabbit whole IgG horseradish peroxidase conjugated antibody (Sigma). The final reactions were developed with tetra-methyl-benzidine substrate (BD Biosciences), stopped with 2 N H_2_SO_4_, and read at 450 nm in a Multiskan Ascent apparatus (ThermoElectron).

### Efficacy of immunization and antibody responses assessment

Eight to ten-week old female BALB/c mice (Charles River International) were accommodated (Public University of Navarre registration code ES/31-2016-000002-CR-SU-US) and handled in compliance with the current European, national and local (RD 1201/2005) regulations, following the FELASA and ARRIVE guidelines and with the approval of the UPNA Animal Experimentation Committee and Navarre’s Government. For immunization, mice were inoculated intraperitoneally (IP) with a volume of 0.1 mL of live bacteria or antigenic preparations in PBS. Bacterial suspensions were adjusted by spectrophotometry in PBS (O.D. at 600 nm = 0.150 contains approximately 2 × 10^8^ CFU/mL) and the exact number of CFU in each suspension was retrospectively assessed, by serial dilutions and plating on agar. Efficacy was determined from at least two independent experiments with statistically equivalent controls.

In a first set of experiments, a total of 30 mice (6 groups; 5 mice/group) were IP immunized with HS or bacterin preparations (20 μg protein/mouse), alone or in physical mix with EDA or MEDA (40 μg/mouse). A total of 45 control mice (9 groups; 5 mice/group) were inoculated with: (i) HS-SEwt or B-SEwt (20 μg protein); (ii) PBS; or (iii) EDA or MEDA (40, 100 or 200 μg/mouse). Four weeks later, all mice were challenged IP with the optimal sub-lethal dose of around 2.5 × 10^2^ CFU SE-wt and, 4 days later, the mean (*n* = 5) number of log_10_ CFU/spleen challenging strain was determined [37]. The optimal sub-lethal dose (i.e., the minimal dose able to induce moderate and homogeneous levels of splenic infection in all mice) was estimated in a previous dose–response experiment, where mice (*n* = 5) were IP inoculated with 50, 100, 250, 500 or 1000 CFU/mouse, and log_10_ CFU/spleen determined 4 days later.

A similar immunization-challenge murine model (but using 4 mice/group) was applied in a second set of experiments, to determine the efficacy of biotinylated bacterins (20 μg/mouse) bound to EDAvidin (75 μg/mouse) (i.e. BEDA-SEΔ*waaL* and BEDA-SEΔ*gal*, respectively). Besides the above-mentioned controls, live SEΔ*waaL* or SEΔ*gal* live mutants (1 × 10^4^ CFU/animal, IP) were included. Persistence of SEΔ*waaL* at the end of the experimental period was distinguished from that of SE-wt challenging strain by double plating in agar and agar supplemented with chloramphenicol (20 mg/L). Persistence of SEΔ*gal* was not determined since this mutant was cleared from spleens within 2 weeks post-inoculation, as verified in the virulence assay (Additional file 1: Table S1).

Just before challenge, mice sera were analysed for *Salmonella* specific immunoglobulin (IgG + IgM and IgG2a/IgG1) quantification by indirect ELISA, using HS extracts (HS-SEwt for IgG + IgM or homologous HS extract for IgG2a/IgG1 isotype determination) as coating antigens and horseradish anti-mouse IgG and IgM (H + L) (InmunoPure, Pierce) or anti-mouse IgG1 or IgG2a (Nordic Immunological) as conjugates. Positive control sera from mice experimentally infected with SE-wt, and negative control sera from animals inoculated with PBS were included in each ELISA plate. Serum titre was defined as the reciprocal of the highest serum dilution showing a mean O.D. value equal to or higher than that of the negative control sera (*n* = 4) obtained from mice inoculated with PBS, plus 3 times the SD. Titers of IgG + IgM were expressed as the mean and SD of individual log_10_ titre obtained; and the Th1/Th2 balance, as the mean and SD of individual log_10_ IgG2a/log_10_ IgG1 ratio [38].

### Statistical analysis

Kolmogorov-Smirnov test was first applied to assess the normal distribution of data. Then, means were statistically compared by a one-way ANOVA test, followed by the Fisher’s Protected Least Significant Difference (PLSD) test (when four or less groups were compared) or Bonferroni’s test (when more than four groups were compared). Efficacy was determined, from at least two independent experiments with statistically equivalent controls, by statistical comparison of the mean (*n* = 5) log_10_ SE-wt CFU/spleen obtained in immunized vs. control mice. In mice survival assays, the percentage of cumulative survival was calculated by the Kaplan-Meier analysis, and statistically compared by the LogRank (Mantel-Cox) test.

## Results

### Characterization of SEΔ*waaL* and SEΔ*gal* LPS O-PS deficient mutants

**Figure 1:**
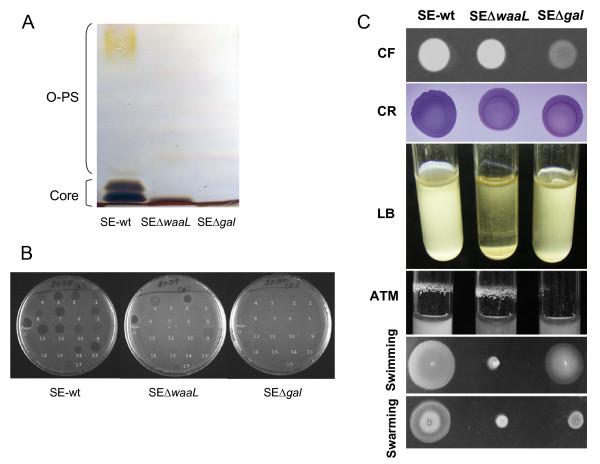
**Phenotypic characterization of* Salmonella*Enteritidis SE∆waaL and SE∆gal mutants compared to SE-wt parental strain.** (**A**) SDS-PAGE with alkaline silver staining of LPS; (**B**) phage susceptibility to the 17 Salmonella Enteritidis Typing Phage collection (named from 1 to 17) and FO bacteriophages; and (**C**) CF: Calcofluor; CR: Congo Red; LB, biofilm formation in static LB rich medium; ATM, biofilm formation in stirring ATM nutrient deficient medium; swimming; and swarming assays.

LPS SDS-PAGE silver stain showed that both rough mutants had an O-PS free LPS, but SEΔ*gal* had a LPS-Core smaller than that of SEΔ*waaL* (Figure 1a), as expected from the mutant design. This difference in LPS-Core size was in agreement with the results obtained in the phage susceptibility assay (Figure 1b), where in contrast to SE-wt, both mutants were resistant to phages that recognize specifically O-PS epitopes (i.e. SETPs 1, 3, 5, 7, 8, and 10–16) belonging to *Podoviridae* and *Siphoviridae* morphotypes [29], but only SEΔ*waaL* retained susceptibility to the *Myoviridae* (i.e. SETPs 2, 4, 9), SETP17 and FO phages that recognize specifically the external LPS-Core epitopes, indicating that this mutant had an intact LPS-Core whereas SEΔ*gal* had a more pronounced defect. Anti-H agglutination indicated that both rough mutants had flagellum antigens. Phenotypic differences in motility and biofilm formation were observed between both rough mutants and with respect to the parental strain SE-wt (Figure 1c). Specifically, the SEΔ*gal* mutant retained partially the flagellum motility, as indicated by the swimming and swarming halos observed, but showed a cellulose production deficiency in the calcofluor binding assay, and also a diminished biofilm formation either in rich LB or in nutrient deficient ATM media (Figure 1c). In contrast, the SEΔ*waaL* mutant lacked motility, but maintained the capability of its parental strain to retain the cellulose on the cell surface and, consequently, to form biofilm in the ATM stirring assay (Figure 1c). Susceptibility to Polymyxin B (Additional file 2: Figure S1-a) showed that the SEΔ*gal* mutant was more susceptible (0.125 μg/mL) to cationic peptides than both SEΔ*waaL* (0.250 μg/mL) and SE-wt (4 μg/mL) strains. Moreover, susceptibility to non-immune serum revealed a drastic reduction in the number of surviving bacteria in both rough mutants compared to strain SE-wt, more marked in SEΔ*gal* (Additional file 2: Figure S1-b). In fact, few colonies of SEΔ*gal* (83 CFU) or SEΔ*waaL* (325 CFU) per million of bacteria resisted the bactericidal effect of serum complement, whereas around 5 × 10^4^ CFU SE-wt were resistant.

Finally, virulence was measured by the ability of bacteria to induce lethality in the infected mice (Additional file 1: Table S1). As expected, all mice inoculated with 2 × 10^3^ CFU of the strain SE-wt died (at day four) after inoculation. However, none (SEΔ*gal*) or only one (SEΔ*waa*L) of the mice inoculated with this dose succumbed at the end of the experiment (2 weeks post-inoculation), indicating that both rough mutants were less (*p* = 0.0027 in LogRank test) virulent than the smooth parental strain SE-wt. Virulence differences were also observed between both rough mutants, since all mice infected with SEΔ*gal*, at any dose, survived to the end of the experimental period without signs of pain or illness, while those inoculated with SEΔ*waa*L showed increased (*p* = 0.035) lethality in cumulative survival analysis when infected at the highest dose, either at day 5 (all the mice inoculated with 2 × 10^7^ CFU/mouse died) or at day 12 (50% of cumulative survival in animals inoculated with 2 × 10^5^ CFU/mouse) (Additional file 1: Table S1). Similarly, highly significant (*p* < 0.005) differences between both rough mutants were observed in the levels of splenic infections found in surviving mice (Additional file 1: Table S1), irrespectively of the infection dose used. Mice inoculated with 2 × 10^3^ or 2 × 10^5^ CFU of SEΔ*gal* were practically (1.3 and 1.6 mean log CFU/spleen, respectively) cleared from infection at week 2, and those infected at a high dose (2 × 10^7^ CFU) were only moderately infected (3.6 mean log CFU). Finally, all surviving mice infected with 2 × 10^3^ or 2 × 10^5^ CFU of SEΔ*waa*L retained more than 4.4 mean log CFU in spleen.

### Characterization of antigen preparations and enhancement of EDA binding to antigen

**Figure 2:**
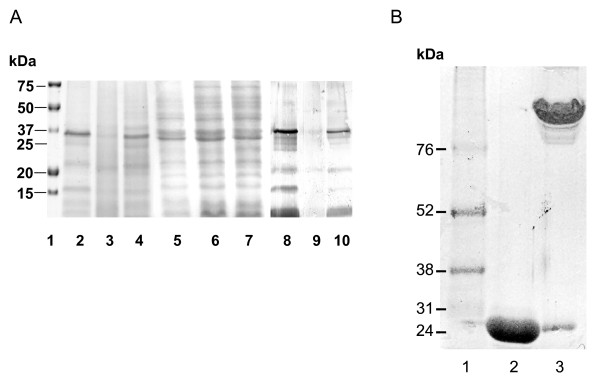
**SDS-PAGE electrophoretic profiles of bacterial proteins from HS and bacterins (Panel A) and EDA based recombinant proteins (Panel B).** (**A**) Coomassie blue (lanes 1–7) and Western-Blot of bacterins using anti-*Salmonella* mouse serum (lanes 8–10). Lane 1: molecular weight marker; lanes 2–4: HS extracts from SE-wt, SEΔ*waaL* and SEΔ*gal*, respectively; lanes 5–7: bacterins from SE-wt, SEΔ*waaL* and SEΔ*gal*, respectively; lanes 8–10: bacterins from SE-wt, SEΔ*waaL* and SEΔ*gal*, respectively; and (**B**) Coomassie blue of EDAvidin denatured by boiling (lane 2) or native (lane 3). Lane 1: molecular weight marker.

The results on protein quantification revealed that HS from rough strains showed an enriched content (20–22% approximately) in comparison with HS-SEwt (around 12%), whereas bacterins from both wild-type and rough strains had a similar protein content (1.1–1.2 mg/mL). The LPS Kdo content was 0.8%, 1.1% and 1.6% in HS-SEΔ*waaL*, HS-SEΔ*gal* and HS-SEwt, respectively. Coomassie and Western-Blot electrophoretic profiles of proteins indicated that HS extracts from rough and wild-type strains displayed similar profiles, including bands of 17 and 21 KDa fimbrial antigens, different porins, and 35–40 KDa outer membrane proteins (Figure 2a). However, bacterins showed a protein spectrum wider than that of HS extracts, including those of high (above 40 KDa, such as 55 KDa flagellin) and low (below 20 KDa) molecular weights, likely eliminated from HS extracts by boiling, autoclaving and/or dialysis (Figure 2a).

According to SDS-PAGE and Western-Blot with anti-His or anti-EDA antibodies, purified EDA and MEDA recombinant proteins had the expected molecular weights (13 and 16 KDa, respectively) [8,39]. Regarding EDAvidin, a 24 KDa band was observed in SDS-PAGE gels (Figure 2b). However, native (unboiled) EDAvidin formed one band likely corresponding to a multimeric form (between 100 and 150 KDa).

**Figure 3:**
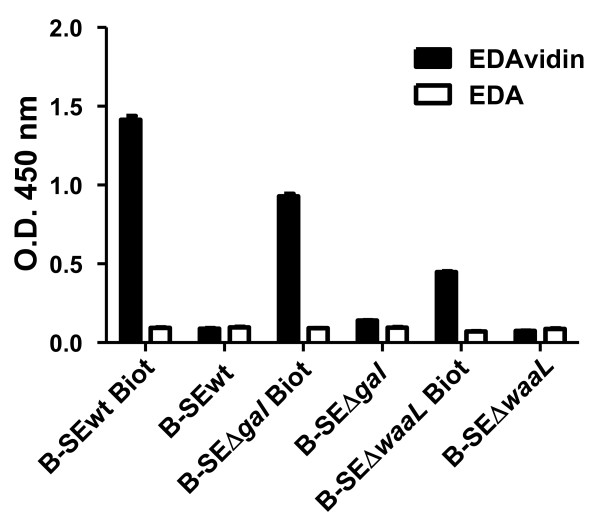
**EDAvidin binding to biotinylated bacterins in ELISA.** ELISA plates coated with biotinylated (Biot) or not biotinylated control (B-SEΔ*waaL*, B-SEΔ*gal* and B-SEwt) bacterins were incubated with EDAvidin or EDA alone (control). Binding was monitored using a rabbit anti-EDA polyclonal antibody and an anti-rabbit whole IgG horseradish-peroxidase-conjugated second antibody. The O.D. values at 405 nm (mean ± SD) are represented.

In order to enhance the binding of EDA to the bacterial antigens, we determined if the capacity of EDA to bind the bacterial cell surface was increased when using EDAvidin and biotinylated bacteria (Figure 3). The results of the ELISA (performed with wells coated with biotinylated or non-biotinylated bacterins) indicated that EDAvidin but not free EDA bound biotinylated bacterins, with increased affinity for biotinylated BEDA-SEΔ*gal* compared to BEDA-SEΔ*waaL* (Figure 3).

Since the biotinylation process involves the binding of Sulfo-NHS-LC biotin to free amines on the bacterial wall, the decreased binding of bacterin SEΔ*waaL* mutant to EDAvidin with respect to SEΔ*gal* or SE-wt could be related to a decreased level of biotinylation of this mutant. The flow cytometry results after CFSE labelling (Additional file 3: Figure S2) revealed that bacterin SEΔ*waaL* had a significantly weaker labeling compared to SEΔ*gal* or SE-wt, in agreement with the results of Figure 3 and the hypothesis proposed.

### EDA/MEDA increases the efficacy of bacterins obtained from LPS O-PS deficient antigens

**Figure 4:**
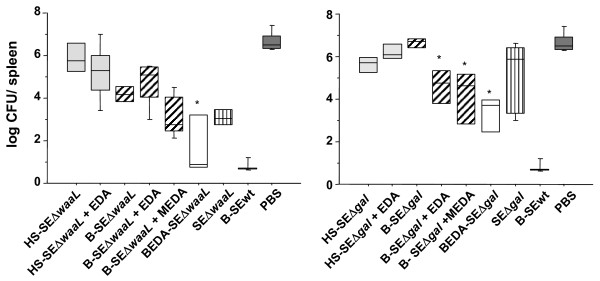
**Protection conferred by antigenic preparations from*Salmonella*Enteritidis rough mutants in BALB/c mice.** Immunizations. (**A**) SEΔ*waaL* immunizations; and (**B**) SEΔ*gal* immunizations. Mice were immunized IP with hot saline (HS) extracts (HS-SEΔ*waaL* and HS-SEΔ*gal*; grey boxes), formalin inactivated bacterins (B-SEΔ*waaL* and B-SEΔ*gal*; grey line boxes), alone, in combination with EDA (+EDA) or MEDA (+MEDA), or as biotinylated bacterins bound to EDAvidin (BEDA-SEΔ*waaL* and BEDA-SEΔ*gal*; white boxes). Control groups of mice (n = 4) received live rough mutants (SEΔ*waaL* or SEΔ*gal*, vertical line boxes), either HS or bacterin obtained from *Salmonella* Enteritidis parental strain (represented as B-SEwt; black boxes) or PBS (black boxes). Four weeks after vaccination, all mice were challenged IP with 2.3 × 10^2^ CFU of *Salmonella* Enteritidis strain 3934 (SE-wt) per animal and the degree of protection expressed as the mean log_10_ CFU/spleen of SE-wt, at day 4 after challenge. Statistical comparisons were performed by ANOVA and Fisher’s PLSD test. * *P* < 0.01 for differences with the corresponding bacterin administered alone, i.e. BEDA-SEΔ*waaL* vs. B-SEΔ*waaL*; and BEDA-SEΔ*gal* or B-SEΔ*gal* plus EDA/MEDA vs. B-SEΔ*gal*.

Mice (*n* = 5) inoculated with increasing doses (40, 100 or 200 μg/mouse) of EDA or MEDA alone did not show either pain signs after inoculation of these compounds or unspecific protection after challenge with SE-wt strain infection, reaching then infection levels of around 7 log CFU/spleen, similar to those reached in unvaccinated controls (Figure 4). Moreover, none of the animals submitted to vaccination in this work presented signs of pain or discomfort and all of them survived up to the end of the experimental period, including those immunized with live mutants.

In the dose–response assay with the virulent strain SE-wt, mice inoculated with 50 or 100 CFU showed inconsistent infections and those inoculated with 500 or 1000 CFU showed saturating infections (Additional file 4: Table S2) with signs of septic shock close to death. In consequence, 2.5 × 10^2^ CFU/mouse was chosen as the optimal sub-lethal dose of challenge in the efficacy experiments.

The effect of EDA on protection was studied in mice (*n* = 5) immunized with HS or bacterins from isogenic rough mutants administered in a simple physical mix with EDA (HS and bacterins) or MEDA (only bacterins) and challenged 4 weeks later with a sublethal dose of SE-wt. In the absence of EDA/ MEDA, vaccination with HS-SEwt or B-SEwt (positive controls of protection) prevented virulent infection, whereas mice unvaccinated (PBS control group) reached around 7 log CFU/spleen (Figure 4). Immunization with HS-SEΔ*waaL*, HS-SEΔ*gal* or B-SEΔ*gal* did not prevent the virulent infection, reaching a mean log CFU of SE-wt in spleen statistically equivalent to that of the PBS control group (Figure 4). In contrast, B-SEΔ*waaL* alone conferred significant protection (*P* = 0.001 vs. PBS control), superior to that conferred by B-SEΔ*gal* (*P* < 0.0001 between both mutants). The combined administration of EDA in preparations with rough HS extracts did not improve protection significantly compared to administration of these antigens alone. Finally, when using bacterins, EDA and MEDA improved significantly (*P* < 0.001) the protection conferred by B-SEΔ*gal* (Figure 4b) but not or only moderately that conferred by B-SEΔ*waa*L (Figure 4a).

### Increased binding of EDA to the antigen enhances protection and antibody production

In order to determine whether a strong binding of EDA to the surface of bacterins might improve immunogenicity, biotinylated bacterins mixed with the EDAvidin (giving rise to the BEDA bacterins) were used for immunization, using live rough mutants as reference. All mice were challenged with the SE-wt strain at week 4 after immunization and the number of SE-wt and SEΔ*waaL* determined in spleen 4 days later. Mice vaccinated with the rough mutants were found less (*P* < 0.05) protected than those immunized with B-SEwt (positive controls). Mice immunized with SEΔ*waaL* mutant retained 3.85 ± 0.21 log_10_ CFU of the mutant in their spleens, revealing the persistence of this mutant throughout the experimental period, with infection levels similar to those observed by this mutant at week 2 (Additional file 1: Table S1). As shown in Figure 4, both BEDA-SEΔ*waaL* and BEDA-SEΔ*gal* preparations improved significantly the levels of protection compared to the bacterin administered either alone (*P* < 0.001) or mixed just with EDA (*P* ≤ 0.03). Strikingly, the efficacy of BEDA-SEΔ*waaL* and BEDA-SEΔ*gal* was, respectively, similar to (*P* > 0.05) and higher than (*P* ≤ 0.01) that conferred by the isogenic live rough mutants. Finally, live rough mutants were less (*P* < 0.05) effective than B-SEwt, whereas BEDA-SEΔ*waaL* conferred a protection statistically equivalent to this positive control (B-SEwt), 2 out 4 mice being free from virulent infection. Therefore, the increased binding of EDA to antigens favoured by streptavidin-biotin interactions resulted in enhanced protection.

In an attempt to initiate a search for an immune correlate with protection, IgG + IgM mean titers and IgG2a/IgG1 ratios were determined in sera obtained just before challenge from mice immunized with bacterins, either physically mixed just with EDA or bound to EDA by streptavidin-biotin (BEDA) interaction. Mice immunized with BEDA-SEΔ*waaL* or B-SEΔ*waaL* + EDA had lower (*P* < 0.01) levels of IgG + IgM than those immunized with the live SEΔ*waaL* mutant (Table 2). In contrast, BEDA-SEΔ*gal* and B-SEΔ*gal* + EDA preparations induced IgG + IgM levels higher (*P* < 0.05) than those induced by the live SEΔ*gal* mutant. The humoral responses to BEDA-SEΔ*waaL* or B-SEΔ*waaL* + EDA were accompanied by an enhanced Th1 response (IgG2a/IgG1 balance between 1.45 ± 0.20 and 1.05 ± 0.20), but this enhancement was not observed in the case of SEΔ*gal* preparations, which induced a response slightly biased towards a Th2 profile (0.74 ± 0.09 and 0.85 ± 0.12 with BEDA-SEΔ*gal* and B-SEΔ*gal* + EDA, respectively).

**Table 2:** Immunoglobulin titres (IgG plus IgM mean log titers and IgG2a/IgG1 log ratios) in mice, measured at week 4 after immunization with bacterins, either physically mixed just with EDA or bound to EDA (EDAvidin) by streptavidin-biotin (BEDA) interaction

**Inoculation group**	**Log_10_ IgM + IgG**	**Log_10_ IgG2a/IgG1 ratio**
	**(mean ± SD) ^a^**	**(mean ± SD) ^a^**
SE-wt live	3.45 ± 0.17	1.18 ± 0.26
SEΔ*waaL* live	3.60 ± 0.73	1.21 ± 0.10
SEΔ*gal* live	2.02 ± 0.45	1.05 ± 0.07
B-SEΔ*waaL* + EDA	2.54 ± 0.38	1.05 ± 0.20
B-SEΔ*gal* + EDA	2.69 ± 0.42	0.85 ± 0.12
BEDA-SEΔ*waaL*	2.77 ± 0.51	1.45 ± 0.20
BEDA-SEΔ*gal*	3.07 ± 0.51	0.74 ± 0.09

^a^ Serum titre was defined as the reciprocal of the highest serum dilution showing a mean O.D. value equal to or higher than that of the negative control sera (*n* = 4) obtained from mice inoculated with PBS, plus 3 times the SD. Mean and SD (*n* = 4) represent the data obtained in triplicate.

## Discussion

Many live attenuated or inactivated vaccines against a variety of pathogens such as *Salmonella* require booster immunizations to attain the expected protection. Since vaccine efficacy may increase with the use of adjuvants, research on adjuvant performance is necessary. In this context, the positive effect of EDA, when administered with non-live *Salmonella* antigenic preparations, was studied in a vaccination-challenge mouse model involving two *Salmonella* Enteritidis rough mutants (SEΔ*waaL* and SEΔ*gal*) differing in the LPS-Core composition. None of these mutants produced LPS O-PS antigen, which may help to distinguish animals vaccinated with these mutants from those infected by field strains. The absence of either (O-PS)-to-LPS assembly enzymes (SEΔ*waaL*) or LPS-Core galactose synthesis enzymes (SEΔ*gal*) led to an intact (in SEΔ*waaL*) or a defective (in SEΔ*gal*) LPS-Core, according to results on SDS-PAGE and susceptibility to SEPTs bacteriophages, as expected from the genetic design of these mutants and previous findings on similar mutants [20,22,40,41]. Functional mutants in the *gal* operon, which includes *gal*E, *gal*T, *gal*K and *gal*M genes, all involved in the synthesis of the LPS-Core galactose [42], have been obtained by inactivation of the *galE* gene [41], but these single-gene mutants may revert to a smooth-LPS by incorporating exogenous precursors of galactose into the biosynthetic pathway, both *in vitro*[41] and *in vivo*[21,43,44]. Thus, the mutant production strategy applied in this work, based on the use of a complete deletion of *gal* operon to produce the SEΔ*gal* mutant, ensured the rough phenotype of this mutant through the blockage of galactose synthesis from endogenous or exogenous sources [44].

When attempting the design of non-live vaccines, HS extracts (enriched in outer membrane components) and formalin-inactivated bacteria (bacterins that retain all the external and internal bacterial antigens) were used as antigens [19,37]. Differences in vaccine efficacy between studies using *Salmonella* Enteritidis HS extracts in mice [19,45] could be explained by differences in bacterial genetic makeup, extract preparation and enrichment methods or immunization vehicles. The HS preparations obtained from the *Salmonella* Enteritidis rough mutants reported here did not protect mice against a virulent challenge, whereas mice immunized with HS-SEwt were protected (100% mouse survival and 80% uninfected spleens), demonstrating the essential role of the LPS O-PS in HS-driven protection. The physicochemical characterization of antigenic preparations confirmed that bacterins had a broader protein spectrum compared to HS extracts. This difference could be related to differences in preparation methods (boiling, ultracentrifugation and dialysis for HS extracts and not bacterins). Bacterins lacking LPS O-PS may be useful vaccines against heterologous *Salmonella* species and serovars, since the rough phenotype has an enhanced immunogenicity of minor antigens, mainly porins and lipoproteins conserved in *Salmonella* serotypes [20]. Interestingly, B-SEΔ*waaL* induced partial protection in mice whereas B-SEΔ*gal* did not confer protection, indicating that a complete LPS-Core could play an essential role.

Once verified that the protection obtained with HS or bacterins from both rough mutants was below that of the B-SEwt, the potential of EDA as immunopotentiator in non-live bacterial vaccines was assessed. Adjuvants such as Freund’s complete or aluminium hydroxide do not appear to improve the immune response against *Salmonella*[46,47], in contrast with polymeric carriers used to adsorb or encapsulate bacterial extracts [19,48]. Alternatively, adjuvants interacting directly with TLRs have successfully immunopotentiated *Salmonella* Enteritidis sub-cellular fractions. Examples are polyinosinic:polycytidylic acid [poly(I:C)] with TLR-3 [49], CpG sequences with TLR-9 [45] and bacterial LPS with TLR-4 [46,50]. Since EDA activates TLR4 favouring viral antigen presentation [6-8,39], mice were immunized in the presence of recombinant EDA (produced in *E. coli*) or MEDA (in transformed plant chloroplast), initially as a simple physical mix with HS extracts (EDA) or bacterins (EDA and MEDA) from *Salmonella* rough mutants.

The effect of EDA in the efficacy of immunizations depended on the type of antigenic preparation (found in bacterins and not HS extracts) and the mutant from which the bacterins were prepared (B-SEΔ*waaL* or B-SEΔ*gal*). No significant adjuvant effects were observed with EDA or MEDA treatments when B-SEΔ*waaL* was administered alone (which already conferred partial protection), but both EDA or MEDA significantly enhanced protection when using B-SEΔ*gal*. This suggests that the size and/or composition of LPS-Core may have affected the affinity for TLR-4 and/or may have regulated the intracellular fate of the antigen in dendritic cells, as demonstrated for LPS O-PS antigen [51]. Possibly, the complete (but not the incomplete) LPS-Core antigen competes with EDA for TLR-4 recognition, so that EDA is not free to interact with this receptor. Alternatively, EDA and MEDA may have a higher affinity for their surface receptors when exposed in absence of the external LPS-Core (i.e. B-SEΔ*gal*).

In search of strategies that would help to enhance the binding of EDA to the antigen, the novel recombinant EDA fused to streptavidin molecule (EDAvidin) allowed a significant binding to biotinylated bacterins. Most likely, biotinylated bacterins decorated with EDAvidin enhanced the targeting of LPS defective bacterins to TLR4 expressing cells, modulating the entry of the antigen and/or its intracellular fate and/or the persistence in dendritic cells [51] to favour the enhancement of the efficacy of these antigenic preparations. This is in line with the significantly improved protection conferred by BEDA-SEΔ*waaL* and BEDA-SEΔ*gal* complexes compared to bacterins alone, reaching levels similar to those obtained with the live rough mutants and, in the case of BEDA-SEΔ*waaL*, the levels conferred by the bacterin B-SEwt positive control. Irrespective of the decreased binding of SEΔ*waaL* to EDAvidin (apparently related to a lower level of biotinylation according to flow cytometry results using CFSE), the protection conferred by each individual bacterin including SEΔ*waaL* increased significantly in the presence vs. absence of EDAvidin. Altogether, these findings demonstrate that EDA in the form of EDAvidin-biotin complexes improves the efficacy of non-live vaccines. Like in previous work [13], increased IgG + IgM levels or a Th1 biased response (according to the IgG2a/IgG1 balance) could not be correlated with the protection conferred by both BEDA preparations (BEDA-SEΔ*waaL* and BEDA-SEΔ*gal*), even though the immune response must have been in both cases sufficiently enhanced to confer significant protection.

Most studies in mice designed to assess *Salmonella* vaccine efficacy use a lethal challenge model. Here, we have used a sub-lethal dose challenge model [19] to preserve animal welfare, yielding information in line with that obtained with the lethal challenge model, since e.g. here live SEΔ*waaL* performed better than SEΔ*gal*, like in previous lethal challenge reports with similar mutants [20]. At the same time, this model allowed the detection of increased protection in mice when EDA or MEDA were administered mixed with B-SEΔ*gal*, and also allowed both the selection of bacterins and not HS from both mutants as *Salmonella* antigen candidates and the detection of enhanced protection with EDAvidin bound to biotinylated B-SEΔ*waaL*.

Although additional work should be done in different natural hosts to determine the true innocuousness and efficacy of BEDA preparations, it is clear that EDA (as EDAvidin) improves the efficacy of rough *Salmonella* bacterins (as biotinylated bacterins) in the mouse model. The association between EDAvidin and B-SEΔ*waaL* bacterin may be considered safe and effective for use as a non-live vaccine, conferring a high protection against virulent infection. Employing this BEDA immunization strategy with O-PS deficient mutants may also help to distinguish (by conventional anti-O-PS or new anti-EDA serological tests) between vaccinated animals and asymptomatically infected carriers, reservoirs of zoonotic infections. Moreover, the use of non-live vaccines avoids the presence of genetically modified microorganisms in farm animals and their subsequent release to environment or food-chain, having an added value for consumers and veterinary use.

## Competing interests

The use of EDAvidin is patent pending.

## Authors’ contributions

LA, VZ, CM and IF carried out the obtaining and characterization of EDA, MEDA, EDAvidin and biotynilated bacterins. BG carried out the construction and genetic characterization of the *Salmonella* mutants. BSR, VG, PMM and XDA carried out the microbiological characterization of the mutants, the obtaining and characterization of the *Salmonella* antigens, the immune response and protection assays in mice, and the statistical analysis. IL and DDA participated in the draft of the manuscript. BA, JJL and MJG conceived the study, participated in its design, coordination, and interpretation of the results, and wrote the manuscript. All authors read and approved the final manuscript.

## Supplementary Material

Additional file 1: Table S1 Virulence of SEΔgal and SEΔ*waaL* in BALB/c mice. Click here for file

Additional file 2: Figure S1 Susceptibility of *Salmonella* Enteritidis parental and mutant strains to cationic peptides and non-immune human serum. (A) Minimal Bactericidal Concentration (MBC) to Polymyxin B; and (B) Susceptibility to conventional human serum, with respect to heat-inactivated serum. Results are expressed as the mean and SD (*n* = 6) of Polymyxin B concentration (μg/mL) at which bacteria were not recovered (A); and log_10_ CFU of viable bacteria in fresh serum per million of viable bacteria surviving in heat-inactivated serum (B). Statistical differences (*P* < 0.01) were found by Fisher’s PLSD test between SE-wt and each rough mutant. Click here for file

Additional file 3: Figure S2 Flow cytometry of bacterins B-SEΔgal, B-SEΔ*waaL* and B-SEwt labeled with carboxyfluorescein succinimidyl ester (CFSE). Unlabeled SE-wt bacterins (B-SEwt) were used as negative control. Click here for file

Additional file 4: Table S2 Dose–response assay with *Salmonella* Enteritidis 3934 (SE-wt) strain in mice. Click here for file
