# Supplementary material for: The extradomain a of fibronectin enhances the efficacy of lipopolysaccharide defective Salmonella bacterins as vaccines in mice
Source: Vet Res. 2012 Apr 19;43(1):31. doi: 10.1186/1297-9716-43-31 (PMC3418186; doi:10.1186/1297-9716-43-31)
Supplement: Additional file 1: Table S1 — Virulence of SEΔgal and SEΔwaaL in BALB/c mice. [file 1297-9716-43-31-S1.doc]

**Table S1** **Virulence of SEΔ*gal* and SEΔ*waa*L in BALB/c mice.**

^a^ Number of survivors out of the total number of mice (*n* = 5) two weeks after intraperitoneal inoculation with the indicated dose of infection by SEΔ*waaL* or SEΔ*gal* rough mutants. Mice inoculated with 2 × 10^3^ CFU of the smooth parental SE-wt were used as reference.

| Bacterial strain | Dose of infection (CFU/mice) | Survivors/Totals ^a^ | log CFU/spleen (mean ± SD) ^b^ |
| --- | --- | --- | --- |
| SEΔ*waaL* | 2 × 10^3^ | 4/5 | 4.9 ± 1.8 |
|  | 2 × 10^5^ | 3/5 | 4.4 ± 0.2 |
|  | 2 × 10^7^ | 0/5 | N.D. |
| SEΔ*gal* | 2 × 10^3^ | 5/5 | 1.3 ± 0.9* |
|  | 2 × 10^5^ | 5/5 | 1.6 ± 0.1** |
|  | 2 × 10^7^ | 5/5 | 3.6 ± 1.4 |
| SE-wt | 2 × 10^3^ | 0/5 | N.D. |

^b^ Levels of splenic infections in surviving mice, expressed as the mean ± SD of individual log_10_ CFU/spleen, and statistically compared by Fisher’s PLSD test: **P*< 0.005 and ***P*< 0.0005 indicate significant differences between both mutants, inoculated with the bacterial dose indicated. N.D.: not determined, since all the mice of the group died.
