# Supplementary figures and images for: The extradomain a of fibronectin enhances the efficacy of lipopolysaccharide defective Salmonella bacterins as vaccines in mice
Source: Vet Res. 2012 Apr 19;43(1):31. doi: 10.1186/1297-9716-43-31 (PMC3418186; doi:10.1186/1297-9716-43-31)

**A**

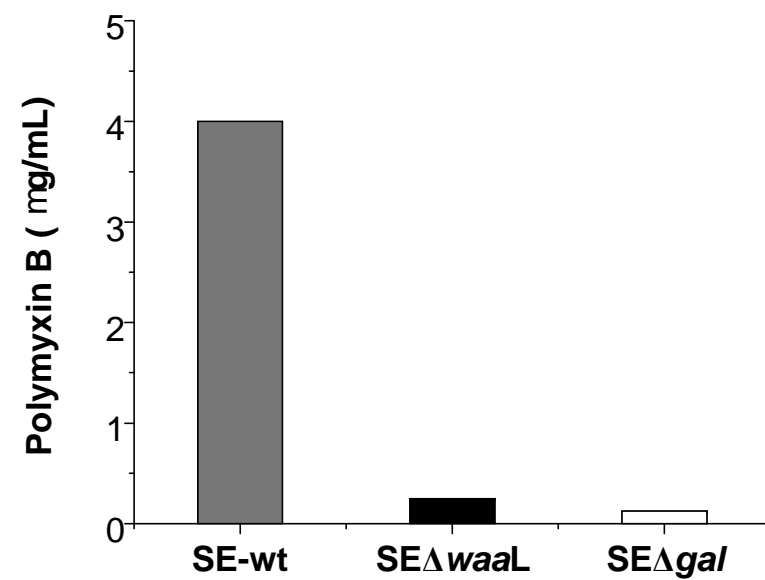

**B**

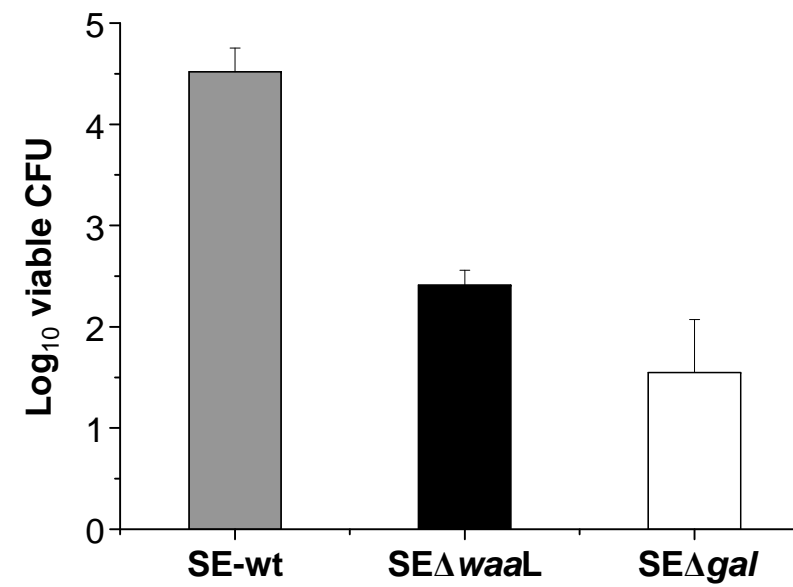

**Figure S1**

Supplement: Additional file 2: Figure S1 — Susceptibility of Salmonella Enteritidis parental and mutant strains to cationic peptides and non-immune human serum. (A) Minimal Bactericidal Concentration (MBC) to Polymyxin B; and (B) Susceptibility to conventional human serum, with respect to heat-inactivated serum. Results are expressed as the mean and SD (n = 6) of Polymyxin B concentration (μg/mL) at which bacteria were not recovered (A); and log10 CFU of viable bacteria in fresh serum per million of viable bacteria surviving in heat-inactivated serum (B). Statistical differences (P < 0.01) were found by Fisher’s PLSD test between SE-wt and each rough mutant. [file 1297-9716-43-31-S2.pdf]

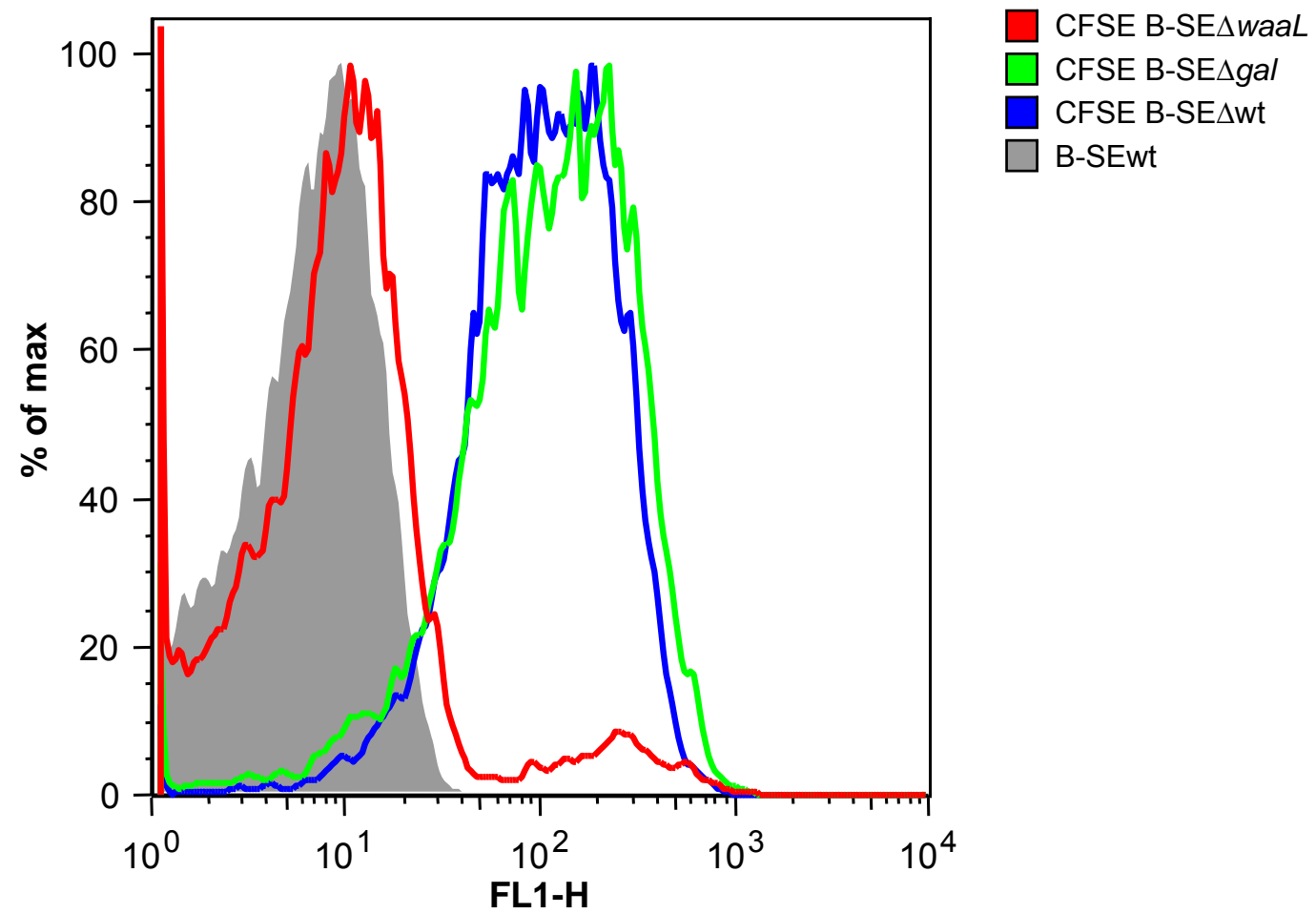

**Figure S2**

Supplement: Additional file 3: Figure S2 — Flow cytometry of bacterins B-SEΔgal, B-SEΔwaaL and B-SEwt labeled with carboxyfluorescein succinimidyl ester (CFSE). Unlabeled SE-wt bacterins (B-SEwt) were used as negative control. [file 1297-9716-43-31-S3.pdf]
