# Supplementary material for: The extradomain a of fibronectin enhances the efficacy of lipopolysaccharide defective Salmonella bacterins as vaccines in mice
Source: Vet Res. 2012 Apr 19;43(1):31. doi: 10.1186/1297-9716-43-31 (PMC3418186; doi:10.1186/1297-9716-43-31)
Supplement: Additional file 4: Table S2 — Dose–response assay with Salmonella Enteritidis 3934 (SE-wt) strain in mice. [file 1297-9716-43-31-S4.doc]

**Table S2 Dose-response assay with *Salmonella* Enteritidis 3934 (SE-wt) strain in mice.**

| Dose of infection (CFU/mice) ^a^ | log CFU/spleen (mean ± SD) ^b^ |
| --- | --- |
| 0.5 × 10^2^ | 3.31 ± 2.59 ^c^ |
| 1 × 10^2^ | 5.82 ± 1.29 ^c^ |
| 2.5 × 10^2^ | 6.59 ± 0.43 |
| 5 × 10^2^ | 6.83 ± 0.05 ^d^ |
| 1 × 10^3^ | 8.34 ± 0.54 ^d^ |

^a^ BALB/c mice were intraperitoneally inoculated with different doses of *Salmonella* Enteritidis 3934 virulent strain.

^b^ The mean and SD (*n*= 5) of log_10_ CFU/spleen of the SE-wt strain was determined 4 days after inoculation.

^c^ mice were inconsistently infected.

^d^ mice showed signs of septic shock.
